# Safety and immunogenicity of ChAdOx1 nCoV-19 (AZD1222) vaccine in adults in Kenya: a phase 1/2 single-blind, randomised controlled trial

**DOI:** 10.12688/wellcomeopenres.19150.1

**Published:** 2023-04-24

**Authors:** Mainga Hamaluba, Samuel Sang, Benedict Orindi, Irene Njau, Henry Karanja, Naomi Kamau, John N. Gitonga, Daisy Mugo, Daniel Wright, James Nyagwange, Bernadette Kutima, Donwilliams Omuoyo, Mwaganyuma Mwatasa, Caroline Ngetsa, Charles Agoti, Stanley Cheruiyot, Amek Nyaguara, Marianne Munene, Neema Mturi, Elizaphan Oloo, Lynette Ochola-Oyier, Noni Mumba, Cynthia Mauncho, Roselyne Namayi, Alun Davies, Benjamin Tsofa, Eunice W. Nduati, Nadia Aliyan, Kadondi Kasera, Anthony Etyang, Amy Boyd, Adrian Hill, Sarah Gilbert, Alexander Douglas, Andrew Pollard, Philip Bejon, Teresa Lambe, George Warimwe, COV004 Vaccine Trial Group

**Affiliations:** 1 KEMRI-Wellcome Trust Research Programme, Kilifi, Kenya; 2 Oxford Vaccine Group, University of Oxford, Oxford, England, UK; 3 Centre for Tropical Medicine & Global Health, University of Oxford, Oxford, England, UK; 4 Ministry of Health, Nairobi, Kenya; 5 The Jenner Institute, University of Oxford, Oxford, England, UK; 6 Pandemic Sciences Institute, University of Oxford, Oxford, England, UK

**Keywords:** COVID-19, vaccine, ChAdOx1-nCoV-19, Kenya

## Abstract

**Background:** There are limited data on the immunogenicity of coronavirus disease 2019 (COVID-19) vaccines in African populations. Here we report the immunogenicity and safety of the ChAdOx1 nCoV-19 (AZD1222) vaccine from a phase 1/2 single-blind, randomised, controlled trial among adults in Kenya conducted as part of the early studies assessing vaccine performance in different geographical settings to inform Emergency Use Authorisation.

**Methods:** We recruited and randomly assigned (1:1) 400 healthy adults aged ≥18 years in Kenya to receive ChAdOx1 nCoV-19 or control rabies vaccine, each as a two-dose schedule with a 3-month interval. The co-primary outcomes were safety, and immunogenicity assessed using total IgG enzyme-linked immunosorbent assay (ELISA) against SARS-CoV-2 spike protein 28 days after the second vaccination.

**Results:** Between 28 ^th^ October 2020 and 19 ^th^ August 2021, 400 participants were enrolled and assigned to receive ChAdOx1 nCoV-19 (n=200) or rabies vaccine (n=200). Local and systemic adverse events were self-limiting and mild or moderate in nature. Three serious adverse events were reported but these were deemed unrelated to vaccination. The geometric mean anti-spike IgG titres 28 days after second dose vaccination were higher in the ChAdOx1 group (2773 ELISA units [EU], 95% CI 2447, 3142) than in the rabies vaccine group (61 EU, 95% CI 45, 81) and persisted over the 12 months follow-up. We did not identify any symptomatic infections or hospital admissions with respiratory illness and so vaccine efficacy against clinically apparent infection could not be measured. Vaccine efficacy against asymptomatic SARS-CoV-2 infection was 38.4% (95% CI -26.8%, 70.1%; p=0.188).

**Conclusions:** The safety, immunogenicity and efficacy against asymptomatic infection of ChAdOx1 nCoV-19 among Kenyan adults was similar to that observed elsewhere in the world, but efficacy against symptomatic infection or severe disease could not be measured in this cohort.

**Pan-African Clinical Trials Registration:** PACTR202005681895696 (11/05/2020)

## Introduction

Coronavirus disease 2019 (COVID-19) remains a major threat to global public health. As of December 2022, there were over 600 million COVID-19 cases, including over 6.5 million deaths reported to the World Health Organization (WHO). However, the true level of transmission of COVID-19 is underestimated because a significant number of people who are asymptomatic or have mild infections go unreported as they do not seek healthcare. Mathematical modelling based on data from 185 countries and territories does indeed suggest a much higher burden, with COVID-19 mortality during 2020-2021 estimated at 19.8 million ^1^. High levels of severe acute respiratory syndrome coronavirus 2 (SARS-CoV-2) exposure have been reported in populations in Africa with a significant clinical burden. However, a large proportion of the infected individuals show no clinical symptoms following SARS-CoV-2 infection, such as in Kenya where over 90% of infections have been asymptomatic ^2^, and admission rates to government hospitals have not shown substantial increases ^3^. This contrasts the situation in populations outside Africa where clinical SARS-CoV-2 infections are more common and, in the absence of mitigation measures, hospitals have been overwhelmed. Nevertheless, there are indications of excess mortality in older age groups ^4^. 

A range of approved COVID-19 vaccines have proven highly effective against COVID-19 hospitalisation and death in diverse geographical settings including South Africa ^5^. However, COVID-19 vaccine coverage in Africa remains poor. While many countries in Europe have deployed multiple additional vaccine doses to boost immunity from the initial primary series of vaccination, less than 30% of the population in Africa had been fully vaccinated with the initial primary series as of December 2022 with an even smaller proportion (~3%) receiving booster doses ^5^. Further, no clinical trial reports on the safety and immunogenicity of currently available COVID-19 vaccines on African populations are available outside of South Africa, with data post Emergency Use Authorisation being few and limited to healthcare workers ^6, 7^.

Here, we report safety and immunogenicity data from adults in coastal Kenya included in a phase 1/2 randomised controlled trial of ChAdOx1 nCoV-19 (AZD1222; COV004 trial) as part of the early studies assessing vaccine performance in different geographical settings to inform Emergency Use Authorisation ^8, 9^. The ChAdOx1 nCoV-19 vaccine developed at the University of Oxford consists of the replication-deficient simian adenovirus vector ChAdOx1 encoding the full-length SARS-CoV-2 spike protein ^8, 9^. ChAdOx1 nCoV-19 was granted Emergency Use Authorisation by regulators including the WHO following promising efficacy results from trials in the United Kingdom, Brazil and South Africa ^8, 9^. Subsequently, due to cold-chain requirements, scale of manufacture, availability and cost, ChAdOx1 nCoV-19 has been one of the most commonly used COVID-19 vaccines with widespread roll-out in low-and middle-income countries (LMICs) including Kenya ^5^. Data on safety and immunogenicity in Africa are important to support use.

## Methods

### Study design

We conducted a phase 1/2 single-blinded, randomised, controlled trial with the primary objective of evaluating the safety and immunogenicity of ChAdOx1 nCoV-19 vaccine in comparison to rabies vaccine as a control in adults aged ≥18 years. Vaccines were administered as two doses three months apart and the subjects followed up for 1 year from first vaccination. Enrolment in the phase 1 component of the trial was aimed at a first evaluation of the safety, tolerability and immunogenicity of the ChAdOx1 nCoV-19 (n=20) in comparison to the rabies control vaccine (n=20) in healthy adults in Kenya aged 18-55 years. Progression to the phase 2 trial among healthy adults aged ≥18 years (n=180 per vaccine arm) followed safety reviews of phase 1 data accrued up to 28 days post-vaccination by an independent international data and safety monitoring board (DSMB; *Extended data* ^10^). This study is reported in line with the Consolidated Standards of Reporting Trials (CONSORT) checklist ^10^.

### Participants

Participants were recruited following extensive community engagement activities, modified from usual practice to comply with existing government physical distancing directives. Recruitment posters and physically distanced door to door engagement were used to give information to prospective participants and information about the trial also provided through interactive radio, virtual seminars, and other media interviews by the study team. An animated video showing how the vaccine was made and some study procedure was developed and shared through social media platforms to reach a wider population. Written informed consent was obtained from all participants after which they were screened for eligibility. Recruitment was within Kilifi and Mombasa counties. The screening visit included an assessment of good health, a full medical history, clinical examination, and laboratory investigations. Participants with significant acute or chronic diseases such as congenital heart disease, renal failure, hepatitis, HIV, Hepatitis B and C, chronic respiratory conditions, hypertension, diabetes amongst others were excluded from the trial. We also excluded females who were pregnant or breastfeeding. Similarly, female participants were required to use effective contraception for 30 days prior to vaccination. Potential participants with a history of laboratory-confirmed or suspected SARS-CoV-2 infection (new onset fever and a cough or shortness of breath in the 30 days preceding screening) were excluded. Ethical and regulatory approvals were obtained from the Kenya Medical Research Institute (KEMRI) Scientific and Ethics Research Unit (KEMRI/SERU/CGMR-C/CSC197/4024 on 24 ^th^ August 2020), the Kenya Pharmacy and Poisons Board (ECCT/20/05/01 on 8 ^th^ September 2020), the National Commission for Science, Technology and Innovation (NACOSTI/P/22/20461 on 11 ^th^ September 2020) and the University of Oxford Tropical Research Ethics Committee (Reference 33-20 on 11 ^th^ June 2020). The trial was registered with the Pan African Clinical Trials Registry (PACTR202005681895696) on 11 ^th^ May 2020.

### Randomisation and masking

Participants were randomised 1:1 to receive either the ChAdOx1 nCoV-19 vaccine or the rabies vaccine. The rabies (Verorab) vaccine was used as the control vaccine. The randomisation list was generated using Stata (StataCorp, College Station, TX) and random allocation using REDCap and access restricted to the unblinded team. Participants were randomly allocated to one of the two vaccine arms as per the computer-generated randomisation schedule. The participants, all personnel assessing the safety endpoints and the laboratory team responsible for sample processing and immunological assays were blinded to vaccine allocation. The vaccines were prepared out of sight of the participant and syringes masked with an opaque object/material until ready for administration to ensure blinding. The control vaccine was also administered on the same site (intramuscularly) as the investigational product to maintain blinding. Following the Kenyan government roll-out of vaccination against COVID-19 participants were unblinded. All unblinded participants were kept in the study regardless of the COVID-19 vaccines received.

### Procedures

The ChAdOx1 nCoV-19 vaccine used in the trial was manufactured by Advent s.r.l. (Pomezia, Italy) and COBRA Biologics (Keele, UK) in accordance with current Good Manufacturing Practice and approved by the UK Medicines and Healthcare Regulatory Agency as described previously ^8^. ChAdOx1 nCoV-19 was used at the standard dose of 5 × 10 ^10^ virus particles. VERORAB (Sanofi) rabies vaccine was purchased locally and used at the standard dose of 0.5mL as per the manufacturer’s summary product characteristics. Both vaccines were administered intramuscularly into the non-dominant deltoid. Participants were considered enrolled in the study once vaccinated. Following vaccination participants were observed for at least one hour post-vaccination to assess for adverse events. Vital signs were recorded as well as axillary temperature and solicited and unsolicited adverse events (AEs) in a diary card. Local solicited AEs assessed in the trial included: pain at injection site, tenderness, redness, warmth, itchiness, swelling and induration. In addition, the systemic solicited AEs included: fever, chills, joint pains, muscle pains, fatigue, malaise, nausea and headache. Before leaving the clinic on vaccination day, the participants were taught how to complete the diary cards and document the timing and severity of the solicited and unsolicited AEs. Participants were also given emergency contact numbers (24/7) for the study team in the event of an emergency or inquiries. The study team contacted the participants daily from the 1 ^st^ to the 6 ^th^ day post-vaccination to remind them to complete the diaries and collect any information on new symptoms or medication. On day 7 after vaccination, the participants would attend the clinic and return the paper diaries.

### Surveillance for COVID-19

Participants underwent nasopharyngeal swabbing at routine pre-specified visits whether symptomatic or not and were asked to contact the study team for a series of swabs and assessments if they had any symptoms of COVID-19 as mandated by the prevailing government directives. Where attendance was not immediately possible participants would be swabbed at the earliest opportunity.

### Outcomes

The co-primary outcomes of the trial were vaccine immunogenicity (seroconversion) as measured by IgG ELISA against SARS-CoV-2 spike protein 28 days after the second dose of vaccine, and safety as assessed by occurrence of adverse events. Secondary outcomes included assessment of IgG titres and their durability, T cell response as measured by *ex vivo* interferon-γ enzyme-linked immunospot (ELISpot) assay and vaccine efficacy against COVID-19.

### Statistical analysis

The phase 1 trial was designed to include 40 participants to obtain early descriptive safety data on common, important adverse events following vaccination with two doses of 5×10 ^10^ vp ChAdOx1 nCoV-19 (n=20) or two doses of control rabies vaccine (n=20). The primary endpoint for the phase 2 trial (n=180 per group) was seroconversion as measured by IgG ELISA against SARS-CoV-2 spike protein 28 days after the second dose of ChAdOx1 nCoV-19 vaccine. A sample size of 200 per vaccine group (combined phase 1b and phase 2) would allow detection of at least 70% seroconversion with <5% error margin. Three analysis populations were defined. The intention-to-treat (ITT) population comprised all randomised participants who received a study vaccine and that had at least one post-vaccination blood sample. The per-protocol (PP) population included randomised participants who had a blood sample at baseline and 28 days (+ 3 days) post-vaccination, and for whom the eligibility criteria were correctly applied. The safety population included all subjects who received a study vaccine (i.e. ITT). Participants were analysed according to the treatment they received. Participants were censored in the analysis of efficacy endpoints at the time of their unblinding and vaccination. However, they contributed to exploratory immunogenicity analyses which are descriptive or observational according to the vaccines they received.

Safety data were summarised as the number of participants that experienced any event post-vaccination in the vaccine group and as a percentage of the total safety population within the vaccine group. For each solicited local and systemic reaction, a relative risk with 95% confidence interval (CI) was presented. This analysis included all AEs up to 28 days post-vaccination. Intra-individual numbers of reactions were compared between vaccine groups using Wilcoxon’s ranksum test. This was done for solicited local and systemic reactions combined, and separately for local and systemic reactions. Similar analysis was repeated for unsolicited reactions. Proportion of participants in each vaccine group reporting any local reaction were compared using the chi-squared test and the difference in proportions with 95% CIs was presented. This was repeated for systemic reactions. All SAEs were described in detail for each participant.

We summarised the number and percentage of participants who seroconverted together with their Wilson-type 95% CIs. Seroconversion rates were compared between the two vaccine groups using relative risk (RR). This was done at 28 days after second dose vaccination, at day 182 and day 365. For the primary timepoint (i.e. at 28 days after the second dose of vaccine), relative risks adjusted for effect of sex, body mass index (BMI) and age at enrolment was estimated using a log binomial model. 28 days after second dose vaccination, GMT and GMT ratio for ChAdOx1 nCoV-19 vaccine to rabies vaccine, together with their 95% CIs, were estimated. Log transformed anti-Spike IgG response at multiple time points were analysed using a linear mixed effect model, with participant as a random effect. Fixed effects included vaccine group, day of visit, a vaccine group-by-day of visit interaction term, baseline IgG response values, sex, BMI and age (categorised in tertiles). Cox regression was used to estimate vaccine efficacy against virologically confirmed SARS-CoV-2 infection. Only events that occurred more than 14 days after the second vaccination dose were included in efficacy evaluations. All analyses were performed using Stata 15.1 (StataCorp, College Station, TX) and GraphPad Prism v9 used to generate graphs.

### Role of the funding source

The funders of the study had no role in the study design, data collection, data analysis, data interpretation, or writing of the report. All authors had full access to all the data in the study and had final responsibility for the decision to submit for publication.

## Results

**Figure 1.**
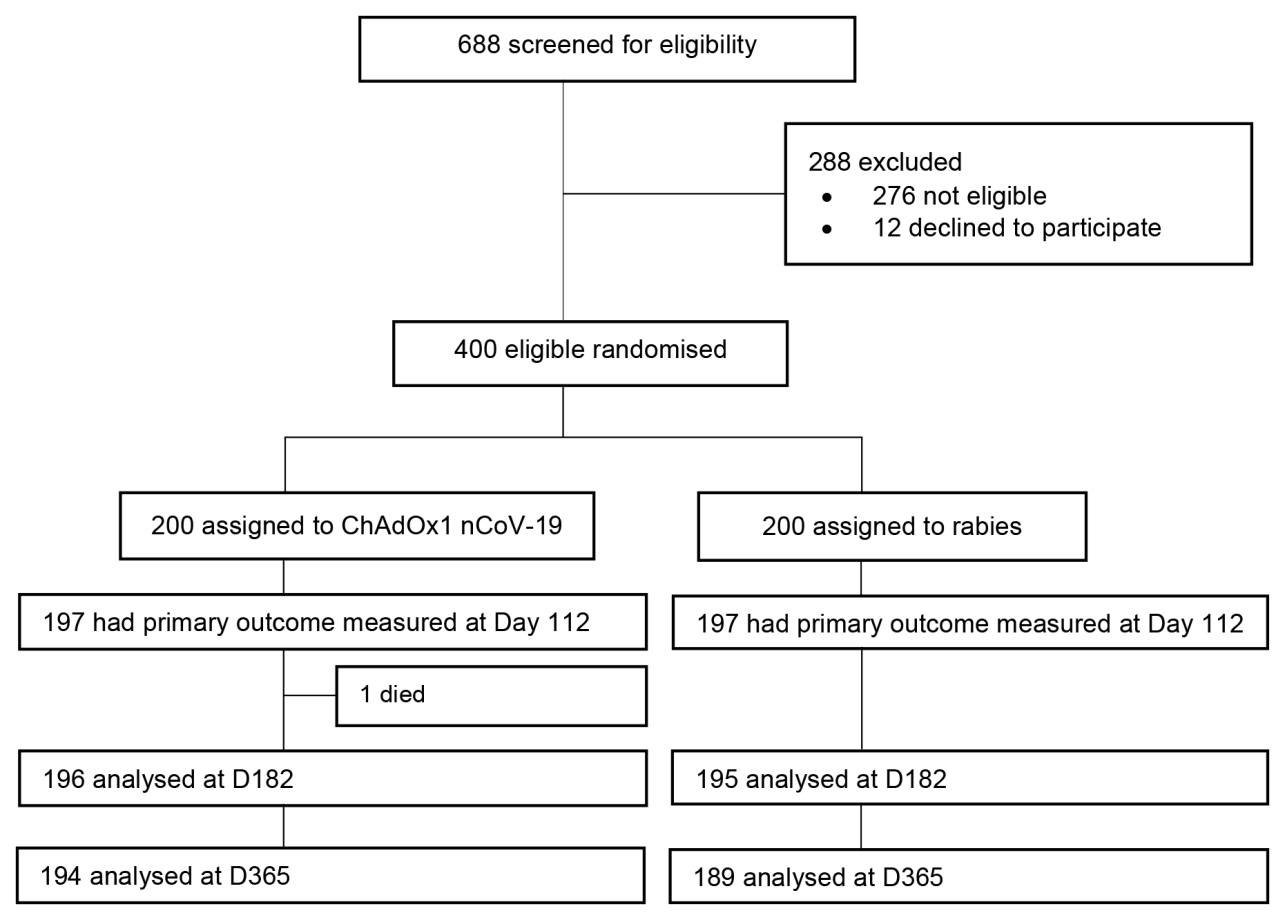
CONSORT diagram. The per-protocol population comprised randomized participants who had a blood sample at baseline and 28 days after second dose vaccination, and for whom the eligibility criteria were correctly applied. The main reason for withdrawal or not completing vaccination regimen was relocation outside of the study area. One participant died during follow-up, unrelated to vaccination. Ten participants in the rabies group were censored by day 112, additional 9 participants by day 182 and a further 11 participants by day 365.

Between 28 ^th^ October 2020 and 19 ^th^ August 2021, 688 volunteers were screened ( Figure 1). We excluded 276 volunteers due to ineligibility while 12 eligible volunteers declined to participate. A total of 400 volunteers were enrolled and randomised for vaccination with ChAdOx1 nCoV-19 (n=200) or rabies vaccine (n=200) and received at least one vaccination ^10^. There were no discontinuations due to protocol deviations. The primary analysis (PP) at day 28 after the second dose vaccination included 197 participants in each vaccine group. Exclusions from the PP were related to migration out of study area (n=2), temporary relocation (n=1) and travel (n=1) for work, and loss to follow-up (n=1). One participant missed their day 112 visit without providing reasons but returned on day 182. Ten participants in the rabies group were censored by day 112 due to unblinding for national COVID-19 vaccination roll-out. Baseline participant characteristics were similar across vaccine groups, although males comprised 81% (n=325) of the study participants. The median age and BMI at enrolment were 29 years and 22.3 kg/m ^2^, respectively ( Table 1). 

**Table 1.** Baseline characteristics of participants enrolled in the study by randomised group.

	ChAdOx1 nCoV-19	Rabies	Combined
**Number enrolled**	200	200	400
**Sex**			
Male	158 (79.0%)	167 (83.5%)	325 (81.3%)
Female	42 (21.0%)	33 (16.5%)	75 (18.8%)
Age in years, median (IQR)	28.5 (24.0 to 36.0)	30.0 (25.0 to 36.0)	29.0 (25.0 to 36.0)
BMI, kg/m ^2^, median (IQR)	22.5 (20.3 to 26.0)	22.3 (20.4 to 24.6)	22.3 (20.3 to 25.2)
Age group, years			
First tertile	62 (31.0%)	77 (38.5%)	139 (34.8%)
Middle tertile	79 (39.5%)	61 (30.5%)	140 (35.0%)
Upper tertile	59 (29.5%)	62 (31.0%)	121 (30.25%)

Data are n (%) or median (interquartile range [IQR])

### Vaccine safety

On average, ChAdOx1 nCoV-19 vaccinees reported more AEs than those vaccinated with rabies vaccine. A total of 358 participants reported 1312 adverse events, with 183 participants in the ChAdOx1 nCoV-19 group reporting 753 AEs and 175 participants in the rabies group reporting 559 AEs ( Table 2). 247 AEs (177 in ChAdOx1 nCoV-19 and 70 in rabies) were deemed definitely related to vaccination, 3 (all in ChAdOx1 nCoV-19) were deemed probably related to vaccination and 316 (145 in ChAdOx1 nCoV-19 and 171 in rabies) were deemed possibly related to vaccination ( Table 2).

**Table 2.** Summary of all adverse events for consented subjects.

	ChAdOx1 nCoV-19	Rabies	Total N=400
**Number of AEs reported**	753	559	1312
**Number of Subjects with AEs [1]**	183	175	358
**Number of SAEs reported**	2	1	3
**Number of Subjects with SAEs [1]**	2	1	3
Number of AEs by Severity *			
Mild	669 (88.8%)	483 (86.4%)	1152 (87.8%)
Moderate	81(10.8%)	74 (13.2%)	155 (11.8%)
Severe	2 (0.3%)	2 (0.4%)	4 (0.3%)
Potentially life-threatening	1 (0.1%)	0 (0.0%)	1 (0.1%)
Subjects with AEs by Severity [2] **			
Mild	181 (90.5%)	171 (85.5%)	352 (88.0%)
Moderate	57 (28.5%)	53 (26.5%)	110 (27.5%)
Severe	2 (1.0%)	3 (1.5%)	5 (1.3%)
Potentially life-threatening	1 (0.5%)	0 (0.0%)	1 (0.3%)
Number of AEs by Relatedness to study product *			
Not related	170 (22.6%)	179 (32.0%)	349 (26.6%)
Unlikely related	258 (34.3%)	139 (24.9%)	397 (30.3%)
Possibly related	145 (19.3%)	171 (30.6%)	316 (24.1%)
Probably related	3 (0.4%)	0 (0.0%)	3 (0.2%)
Definitely related	177 (23.5%)	70 (12.5%)	247 (18.8%)

[1] Subjects who experience one or more AEs or SAEs are counted only once [2] Subjects are counted only once within a particular severity grade or relatedness category *Percentages are based on number of AEs reported for each treatment arm **Percentages are based on N for each treatment arm

ChAdOx1 nCoV-19 vaccinated participants reported significantly more local ( *Extended data* ^10^, Figure S1; p<0.001) and systemic AEs than those in the rabies vaccine group ( *Extended data* ^10^, Table S1 and Figure S1; p=0.001). Local AEs reported were tenderness (98 in ChAdOx1 nCoV-19 and 36 in rabies; RR 2.72, 95% CI 1.84 to 4.11), pain at injection site (74 in ChAdOx1 nCoV-19 and 32 in rabies; RR 2.31, 95% CI 1.51 to 3.62), and induration (5 in ChAdOx1 nCoV-19 and 1 in rabies group; RR 5.0, 95% CI 0.56 to 236.49) ( *Extended data* ^10^, Table S1). Tenderness and pain at injection were more common than any individual local or systemic adverse reactions.

Systemic adverse reactions reported within 7 days after each vaccination were headache, myalgia, fatigue, arthralgia, chills, malaise, nausea and fever. Headache was the most common systemic reaction with both vaccines occurring in 52 (26%) of 200 participants in ChAdOx1 nCoV-19 group and 25 (13%) of 200 participants in rabies group (RR 2.10, 95% CI 1.21 to 3.78). Headache was followed by myalgia which occurred in 35 (18%) of 200 participants in the ChAdOx1 nCoV-19 group and 10 (5%) of 200 participants in the rabies vaccine group (RR 1.47, 95% CI 0.73 to 3.04). The least common systemic adverse reaction was fever which occurred in one participant in each vaccine group. No participant reported a severity grade of 3 or 4 for all solicited local and systemic adverse reactions, except for one participant in the ChAdOx1 nCoV-19 group who had a grade 4 fever.

In total, 79 unique unsolicited adverse reactions (60 in ChAdOx1 nCoV-19 and 53 in rabies vaccine groups) were reported throughout the study ( *Extended data* ^10^, Table S2). The six most common laboratory AEs were hyperkalemia (n=179), increased blood creatinine (n=163), COVID-19 (n=95), leukopenia (n=38), hyperbilirubinemia (n=33) and neutropenia (n=31). There were no statistically significant differences for unsolicited adverse reactions ( *Extended data* ^10^, Table S2; Wilcoxon test p=0.792).

Three serious adverse events were reported throughout the trial, and all were deemed unrelated to vaccination (Listing S1). One serious adverse event was a participant who died of severe head injury with presumed intra-cranial bleeding after falling from a tree at a significant height, >12 feet. Another participant had prolonged hospitalization due to acute gastroenteritis that occurred 15 days after the first vaccination. A third participant suffered a right neck of femur fracture with right hip displacement after falling from a tree.

### Vaccine immunogenicity

The proportion of vaccinees mounting an anti-Spike IgG response (seroconversion) 28 days after the second dose of vaccine was 99.5% (95% CI 97.2 to 99.9) in the ChAdOx1 nCoV-19 group compared with 41.2% (95% CI 34.5 to 48.3) in the rabies vaccine group, yielding a relative risk of seroconversion of 2.42 (95% CI 2.03 to 2.87) ( Table 3). This high seroconversion rate in ChAdOx1 nCoV-19 was maintained at day 182 and day 365 and remained higher than in the rabies vaccine group throughout ( Table 3). Relative risks of seroconversion at day 28 after second dose vaccination remained the same after adjusting for participant characteristics of sex, BMI and age ( *Extended data* ^10^, Table S3). 93 participants – 48 in the ChAdOx1 nCoV-19 and 45 in the rabies groups, respectively – were anti-spike IgG positive at baseline, before vaccination. The rates of seroconversion in the ChAdOx1 nCoV-19 group remained higher than the rabies vaccinated participants on exclusion of these baseline seropositive individuals ( *Extended data* ^10^, Table S4).

**Table 3.** Seroconversion rates at day 112, 182 and 365 in the per-protocol population.

Randomised group	Total (N)	n	% Seroconversion (95% CI)	Relative risk (95% CI)
Day 112				
Rabies	187	77	41.2 (34.5 to 48.3)	
ChAdOx1 nCoV-19	197	196	99.5 (97.2 to 99.9)	2.42 (2.03 to 2.87)
Day 182				
Rabies	174	89	51.1 (43.8 to 58.5)	
ChAdOx1 nCoV-19	196	193	98.5 (95.6 to 99.5)	1.93 (1.66 to 2.23)
Day 365				
Rabies	160	135	84.4 (78.0 to 89.2)	
ChAdOx1 nCoV-19	193	190	98.4 (95.5 to 99.5)	1.17 (1.09 to 1.25)

Data are total number, number seroconverted, percent seroconverted (95% CI) and relative risk (95% CI).

**Figure 2.**
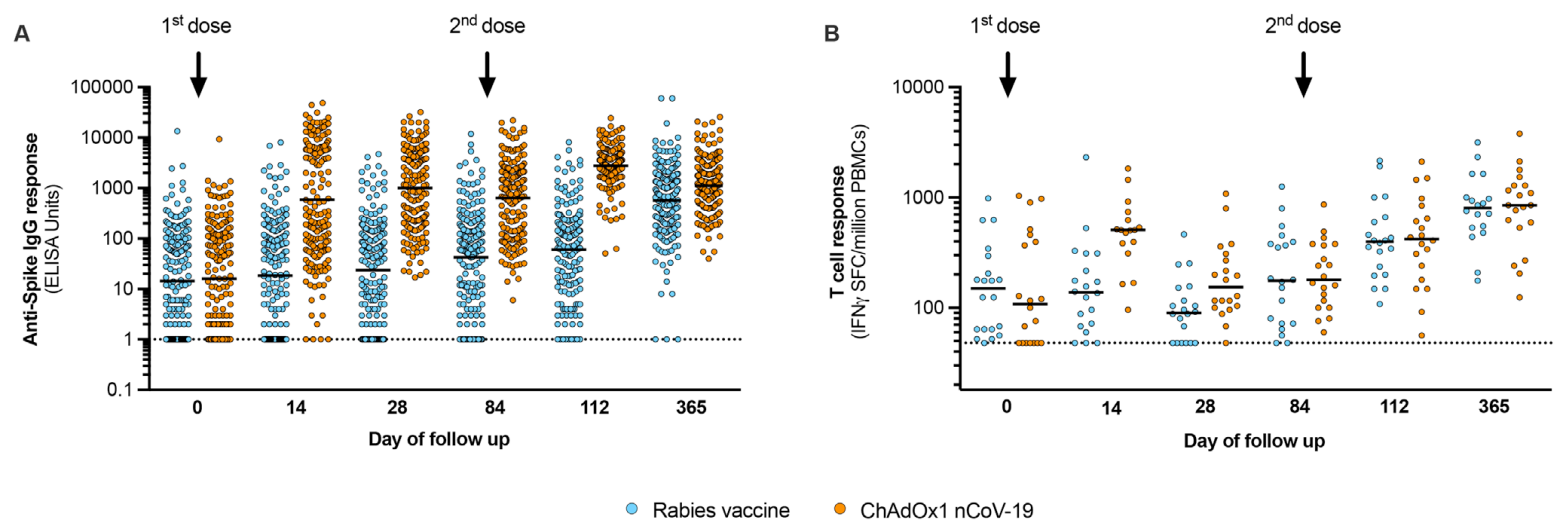
Vaccine immunogenicity. The humoral ( **A**) and cellular responses ( **B**) generated by vaccination are shown for trial participants, with blue markers representing the rabies vaccine group and orange the ChAdOx1 nCoV-19 group. Day 0 represents responses at baseline, before the first vaccine dose. For each group, the second dose was administered on Day 84 as shown. Day 112 represents responses measured 28 days after the second vaccination (primary outcome). Data in ( **A**) represent IgG titres expressed as ELISA units as done for other ChAdOx1 nCoV-19 trials and includes all participants as tabulated in Table 3, with bars representing the geometric mean. Data in ( **B**) represent interferon-γ ELISpot responses against SARS-CoV-2 spike peptides for the first 40 volunteers enrolled in the trial (20 per vaccine arm), with bars representing the median response. PBMCs – peripheral blood mononuclear cells.

We next quantified the anti-spike IgG titres following vaccination, using a quantitative IgG ELISA used for assessing ChAdOx1 nCoV-19 immunogenicity in trial populations in the UK, South Africa and Brazil ^9^. The geometric mean IgG titres (GMT) at 28 days after second dose vaccination were greater in the ChAdOx1 nCoV-19 group (2773, 95% CI 2447 to 3142) than in rabies vaccine group (61, 95% CI 45 to 81) and were maintained through to the end of the 12-month follow-up period ( Figure 2; *Extended data* ^10^, Table S5). The ChAdOx1 nCoV-19 geometric mean IgG titres were within the range generated by ChAdOx1 nCoV-19 vaccination in UK adults ^11^. The T cell response, measured by interferon-γ ELISpot against SARS-CoV-2 spike peptides, peaked at day 14 and showed similar kinetics to other trials of ChAdOx1 nCoV-19 ( Figure 2) ^11^. The median interferon-γ ELISpot response at day 14 for the ChAdOx1 nCoV-19 group was 508 spot-forming cells (SFC) per million peripheral blood mononuclear cells (interquartile range [IQR] 384 to 624), compared with 138 SFC (IQR 70 to 264) for the rabies vaccine group. Both antibody and T cell responses in the rabies vaccine group increased gradually as from day 28 post-vaccination, coinciding with increasing population exposure to SARS-CoV-2 in Kenya ^12^.

### SARS-CoV-2 infections

There were 87 participants with confirmed SARS-CoV-2 infection during the study (ChAdOx1 nCoV-19 = 48, rabies vaccine = 39), eight of whom subsequently had a re-infection (ChAdOx1 nCoV-19 = 2, rabies vaccine = 6). There were no symptomatic, hospitalized or severe cases and vaccine efficacy against clinically apparent infection could not be assessed. All the cases were asymptomatic and were detected on testing their samples collected at the scheduled follow-up visits. Of these 87 cases, 31 (12 in the ChAdOx1 nCoV-19 and 19 in the rabies vaccine group, respectively) occurred ≥14 days after the second dose of vaccine. Given the very few cases, our study was underpowered to detect statistically significant differences in efficacy. A Cox regression model comparing the two vaccines resulted in a vaccine efficacy of 38.4% (95% CI -26.8% to 70.1%; p=0.188), which was comparable to efficacy observed against asymptomatic infection in other populations ^9^.

## Discussion

COVID-19 vaccine coverage in Africa remains poor. Further, while several COVID-19 vaccines have received emergency use approval and been widely deployed, no trials of any COVID-19 vaccine have reported results outside of South Africa ^5^. Data from South Africa include results from randomized controlled trials of ChAdOx1 nCoV-19 ^8, 9^, a recombinant spike protein nanoparticle (Novavax) ^13^ and Ad26.CoV2.S (Janssen) vaccine ^14^. The only other data available are on the post-approval use of the vaccines, including self-reporting of adverse events by healthcare workers in Ethiopia ^7^, and analyses of immune responses in healthcare workers and adults in Nigeria ^6^, Ghana ^6^, Malawi ^15^ and Tunisia ^16^. However, several vaccine trials are now underway in diverse settings across Africa (e.g. ClinicalTrials.gov no. NCT05490108, NCT04934111, NCT05409300 and others).

In this study, we aimed to assess the safety and immunogenicity of ChAdOx1 nCoV-19 in comparison with rabies vaccine among adults in Kenya, adding data to the previous evaluation of the vaccine in trials in South Africa, UK and Brazil ^8, 9^. As observed in these previous trials, ChAdOx1 nCoV-19 was safe with most adverse events being mild or moderate and no serious adverse events related to vaccination ^7^. Vaccination generated strong humoral and cellular immune responses whose kinetics matched those in other populations ^9^. Our data add further support to global regulatory approvals of ChAdOx1 nCoV-19 for a 2-dose regime up to 12 weeks apart.

Through our surveillance for COVID-19 we identified asymptomatic infections but no symptomatic infections. Through our adverse event monitoring, we identified a low prevalence of upper respiratory symptoms and volunteers had decided not to present for testing during these mild symptoms. We identified no hospital admissions with respiratory illness. A possible explanation is that this reflects the epidemiological landscape in Kenya where we observed a predominance of mild and asymptomatic SARS-CoV-2 infections ^2, 17, 18^. Alternatively, it is possible that the general population was avoidant of testing during intercurrent illness, due to the associated stigma and inconvenience of a positive result, and this extended to our trial participants. During assessment of adverse events none of the participants reported taking a COVID-19 test. Though our study was underpowered for efficacy assessment, the efficacy observed against asymptomatic infection was within the range reported in other studies of ChAdOx1 nCoV-19 ^9^.

T cell responses were somewhat lower than those observed in the UK at the peak of 14 days ^11, 19^, though this could be due to lab-to-lab variation in assay performance. Anti-spike IgG antibody responses were within a similar range to those seen in the UK ^11, 20^. Our study sample size precluded assessment of rare adverse events such as thrombosis with thrombocytopenia syndrome (TTS) that has been observed in populations in Europe following adenovirus-vectored COVID-19 vaccines ^21^. ChAdOx1 nCoV-19 has been used in many countries in Africa, but we are aware of no reports of TTS associated with its use on the continent.

Our study had several limitations. First, we predominantly recruited male participants. This is likely primarily due to the study requirement for long-term contraception which goes against cultural norms. Second, due to the prevailing patterns of health seeking behaviour we could not ascertain whether the upper respiratory tract infections reported during follow-up were COVID-19 cases, and there were no symptomatic episodes in any case. Third, due to the long duration of recruitment, trial participants were inevitably exposed to SARS-CoV-2, compromising analysis of vaccine-specific immune responses. This was evident in the gradual increase of anti-spike IgG titres and cellular responses in the rabies vaccine group over time.

We conclude that the vaccine is safe and immunogenic in Kenya, and suitable for ongoing use.

## Data Availability

Harvard Dataverse: Data for: A phase Ib/II single-blinded, randomised, controlled study to determine safety, immunogenicity and efficacy of the candidate Coronavirus Disease (COVID-19) vaccine ChAdOx1 nCoV-19 in adults in Kenya. https://doi.org/10.7910/DVN/L4NX9M ^10^. This project contains the following underlying data: COV004_Analysis_datasets_anon.dta COV004_Analysis_datasets_anon.tab Harvard Dataverse: Data for: A phase Ib/II single-blinded, randomised, controlled study to determine safety, immunogenicity and efficacy of the candidate Coronavirus Disease (COVID-19) vaccine ChAdOx1 nCoV-19 in adults in Kenya. https://doi.org/10.7910/DVN/L4NX9M ^10^. This project contains the following extended data: COV004_analysis_codes.do COV004_Supplementary_Material.pdf COV004_Trial_Dataset_Codebook.pdf COV004_Trial_Dataset_readme.txt COV004_Trial_Protocol.pdf Harvard Dataverse: CONSORT checklist for ‘Safety and immunogenicity of ChAdOx1 nCoV-19 (AZD1222) vaccine in adults in Kenya: a phase 1/2 single-blind, randomised controlled trial’, https://doi.org/10.7910/DVN/L4NX9M ^10^. Data are available under the terms of the Creative Commons Attribution 4.0 International license (CC-BY 4.0)
