# Supplementary material for: Safety and immunogenicity of ChAdOx1 nCoV-19 (AZD1222) vaccine in adults in Kenya: a phase 1/2 single-blind, randomised controlled trial
Source: Wellcome Open Res. 2023 Nov 27;8:182. Originally published 2023 Apr 24. [Version 2] doi: 10.12688/wellcomeopenres.19150.2 (PMC11066537; doi:10.12688/wellcomeopenres.19150.2)
Supplement: Peer review history

# Reviewer report: Reviewer response for version 2

**DOI:** 10.21956/wellcomeopenres.22625.r79286

**Published:** 2024-05-03

**Authors:** Stephen Gordon

**Affiliations:** 1 Malawi-Liverpool-Wellcome Clinical Research Programme, Kamuzu University of Health Sciences, Blantyre, Malawi

This report of a Phase 1/2 study is a model of clear writing and is an excellent example of how to write up an early phase vaccine study.

- The title is clear and explanatory.
- The introduction sets the context well.
- The methods are clear and so are the results. 
- The figures show the data clearly.
- The discussion is straightforward about the study limitations and does not overstate the conclusions.

Is the work clearly and accurately presented and does it cite the current literature?

Yes

If applicable, is the statistical analysis and its interpretation appropriate?

Yes

Are all the source data underlying the results available to ensure full reproducibility?

Yes

Is the study design appropriate and is the work technically sound?

Yes

Are the conclusions drawn adequately supported by the results?

Yes

Are sufficient details of methods and analysis provided to allow replication by others?

Yes

Reviewer Expertise:

Medicine in LMIC.  Respiratory infections and vaccine study including CHIM and experimental medicine.

I confirm that I have read this submission and believe that I have an appropriate level of expertise to confirm that it is of an acceptable scientific standard.

# Reviewer report: Reviewer response for version 2

**DOI:** 10.21956/wellcomeopenres.22625.r70571

**Published:** 2024-05-02

**Authors:** Marianne W. Mureithi

**Affiliations:** 1 Department of Microbiology, University of Nairobi, Nairobi, Kenya

The revised manuscript effectively addresses previous concerns and provides comprehensive insights into the trial's outcomes. I have no major concerns regarding the scientific rigour, data presentation, or conclusions from the findings.

**Specific Comments**

 Introduction Revision:

 The authors have appropriately updated the global COVID-19 burden estimates and clarified the trial's utility in supporting public health decisions. These revisions have enhanced the study's relevance and context, offering readers a clear understanding of its importance in the broader scope of the COVID-19 pandemic response.

**Discussion on Safety Results:**

 Adding a discussion on the safety results in relation to other settings is commendable. Acknowledging the study's limited power to detect rare adverse events provides a balanced view of the findings. It appropriately sets the expectations for the study's contributions to understanding vaccine safety.

**Discussion on Vaccine Immunogenicity:**

 The expanded discussion on vaccine immunogenicity, particularly the T cell response and the observed range of antibody levels, adds depth to the analysis. It enriches the manuscript by providing a comprehensive view of the immune response elicited by the ChAdOx1 nCoV-19 vaccine in the study population. This information is valuable for readers interested in the nuances of vaccine-induced immunity.

**Representativeness and Generalizability:**

 The authors prudently cautioned that the findings from a single community in coastal Kenya might not represent the continent's entire diversity. This acknowledgement is important for contextualizing the study results and underscores the need for further research across different African settings to fully understand the ChAdOx1 nCoV-19 vaccine performance.

**Methodological Rigor:**

 The study's methodological approach is well-designed, and the processes for randomization, masking, and data analysis are robust. The detailed description of these methods enhances the study's transparency and reproducibility, which are crucial aspects of scientific research.

**Public Health Implications:**

 The data supports the study's conclusion that the ChAdOx1 nCoV-19 vaccine is safe and immunogenic in a Kenyan adult population, supporting its ongoing use. The implications for public health policy and vaccine deployment strategies in Africa are significant, particularly in light of the continuing challenges posed by COVID-19.

**Conclusion:**

 The revisions made to the manuscript have adequately addressed the concerns raised in the initial review. The study presents valuable data on the ChAdOx1 nCoV-19 vaccine's safety and immunogenicity in an African population, contributing to the global efforts to combat the COVID-19 pandemic. I commend the authors for their thorough work and have no further comments.

Is the work clearly and accurately presented and does it cite the current literature?

Yes

If applicable, is the statistical analysis and its interpretation appropriate?

Yes

Are all the source data underlying the results available to ensure full reproducibility?

Yes

Is the study design appropriate and is the work technically sound?

Yes

Are the conclusions drawn adequately supported by the results?

Yes

Are sufficient details of methods and analysis provided to allow replication by others?

Yes

Reviewer Expertise:

Immunology and Microbiology

I confirm that I have read this submission and believe that I have an appropriate level of expertise to confirm that it is of an acceptable scientific standard.

# Reviewer report: Reviewer response for version 2

**DOI:** 10.21956/wellcomeopenres.22625.r70570

**Published:** 2023-12-08

**Authors:** Dongming Zhou

**Affiliations:** 1 Tianjin Medical University, Tianjin,, China

The authors fully addressed the reviewers' questions and the current version can be accepted for indexing.

Is the work clearly and accurately presented and does it cite the current literature?

Yes

If applicable, is the statistical analysis and its interpretation appropriate?

Yes

Are all the source data underlying the results available to ensure full reproducibility?

Yes

Is the study design appropriate and is the work technically sound?

Partly

Are the conclusions drawn adequately supported by the results?

Yes

Are sufficient details of methods and analysis provided to allow replication by others?

Partly

Reviewer Expertise:

NA

I confirm that I have read this submission and believe that I have an appropriate level of expertise to confirm that it is of an acceptable scientific standard.

# Reviewer report: Reviewer response for version 1

**DOI:** 10.21956/wellcomeopenres.21229.r68027

**Published:** 2023-10-05

**Authors:** Dongming Zhou

**Affiliations:** 1 Tianjin Medical University, Tianjin,, China

Hamaluba *et al.* conducted a phase 1/2 single-blind clinical trial to evaluate the safety and immunogenicity of ChAdOx1 nCoV-19 among Kenyan adults by using conventional rabies vaccine as a control. The results indicated that the vaccine is safe and immunogenic in Kenya, and suitable for ongoing use. The manuscript is overall well-written and well-structured. Following are the comments and suggestions.

1. The results of immunogenicity test indicated that the rabies vaccine could also stimulate a certain level of spike-specific antibody response and T cell response. Is this possibly related to the fact that all people have been infected with other coronaviruses previously, and other coronaviruses cross-reacts with SARS-CoV-2? Please explain it in the discussion part.
2. Based on the antibody response and T cell response, the immunogenicity of ChAdOx1 nCoV-19 in Kenyan adults is not as good as those in the UK or other populations. Is this possibly related to the pre-existing immunity of ChAdOx1 vector? Have the authors screened the pre-existing immunity of ChAdOx1 vector in the recruited cohorts?
3. Since neutralizing antibody response is crucial for the protection against viral infections, it will be better to measure the neutralizing antibodies to SARS-CoV-2 in the cohorts.
4. In the study, a standard dose of 5 × 10 ^10^ vp was used for immunization. In fact, it will be better to conduct a dose-response experiment to get a more suitable dose firstly for Kenyan adults, since they may have a different immunological background from other populations.

Is the work clearly and accurately presented and does it cite the current literature?

Yes

If applicable, is the statistical analysis and its interpretation appropriate?

Yes

Are all the source data underlying the results available to ensure full reproducibility?

Yes

Is the study design appropriate and is the work technically sound?

Partly

Are the conclusions drawn adequately supported by the results?

Yes

Are sufficient details of methods and analysis provided to allow replication by others?

Partly

Reviewer Expertise:

Anti-infection immunity; novel vaccine development, adenoviral vector design

I confirm that I have read this submission and believe that I have an appropriate level of expertise to confirm that it is of an acceptable scientific standard, however I have significant reservations, as outlined above.

# Response

**Published:** 2023-11-06

**Authors:** George Warimwe

**Affiliations:** KEMRI-Wellcome Trust Research Programme, Kenya

Hamaluba *et al.* conducted a phase 1/2 single-blind clinical trial to evaluate the safety and immunogenicity of ChAdOx1 nCoV-19 among Kenyan adults by using conventional rabies vaccine as a control. The results indicated that the vaccine is safe and immunogenic in Kenya, and suitable for ongoing use. The manuscript is overall well-written and well-structured. Following are the comments and suggestions.

1. The results of immunogenicity test indicated that the rabies vaccine could also stimulate a certain level of spike-specific antibody response and T cell response. Is this possibly related to the fact that all people have been infected with other coronaviruses previously, and other coronaviruses cross-reacts with SARS-CoV-2? Please explain it in the discussion part.

*We agree and have added this point to the discussion as follows: “* *Furthermore, T cell responses were acquired during follow up among Rabies control vaccinees. These T cell responses are likely to be acquired as a result of SARS-CoV-2 transmission in the population, and initial T cell responses prior to vaccination may also be a result of cross-reactivity following circulation of endemic coronaviruses in Kenya (* *https://wellcomeopenresearch.org/articles/5-150* *).  These responses in the control group would further dilute the impression of immunogenicity among ChAdOx1 vaccinees.* *”*

1. Based on the antibody response and T cell response, the immunogenicity of ChAdOx1 nCoV-19 in Kenyan adults is not as good as those in the UK or other populations. Is this possibly related to the pre-existing immunity of ChAdOx1 vector? Have the authors screened the pre-existing immunity of ChAdOx1 vector in the recruited cohorts?

*We did not do this in the current study, but prior work suggest a low prevalence of antibodies to chimpanzee adenoviruses in Kenya (* *https://doi.org/10.1016/j.vaccine.2009.03.080* *).*

1. Since neutralizing antibody response is crucial for the protection against viral infections, it will be better to measure the neutralizing antibodies to SARS-CoV-2 in the cohorts.

*We agree, this would be useful data to report in the future, for now we have assumed that given similar ELISA units in the Kenyan population with populations vaccinated elsewhere that the functional antibody levels would be consistent with this.*

1. In the study, a standard dose of 5 × 10 ^10^ vp was used for immunization. In fact, it will be better to conduct a dose-response experiment to get a more suitable dose firstly for Kenyan adults, since they may have a different immunological background from other populations.

*Dose optimization was conducted in earlier studies in the UK, we agree it may have been optimal to conduct further dose optimization in Kenya but given the context and stage of the pandemic we opted to proceed with a standard dose.*

# Reviewer report: Reviewer response for version 1

**DOI:** 10.21956/wellcomeopenres.21229.r56551

**Published:** 2023-06-16

**Authors:** Marianne W. Mureithi

**Affiliations:** 1 Department of Microbiology, University of Nairobi, Nairobi, Kenya

This study contributes important data on the safety and immunogenicity of the ChAdOx1 nCoV-19 vaccine in an African population. It highlights the vaccine's ability to induce a strong immune response, as evidenced by high seroconversion rates and elevated IgG titres. The findings support using ChAdOx1 nCoV-19 in African countries and provide valuable evidence for vaccine deployment and decision-making.

 Overall, this study contributes significant knowledge on the ChAdOx1 nCoV-19 vaccine's safety and immunogenicity in an African population, supporting its use and providing insights for future research and vaccine implementation.

 The authors could improve the manuscript by addressing the following points:

 Introduction:

1. Lack of recent data: The manuscript references COVID-19 case and death statistics from December 2022, which is outdated considering the current date. Recent data should be used to represent the COVID-19 situation accurately.
2. Inconsistency in COVID-19 severity: The introduction mentions that African populations have a significant clinical burden of SARS-CoV-2 exposure but many infected individuals show no clinical symptoms. However, it also states that hospitals have not shown substantial increases in admission rates. This contrast requires further clarification and supporting evidence.
3. Limited data on vaccine safety and immunogenicity in Africa: The manuscript highlights the lack of clinical trial reports on the safety and immunogenicity of COVID-19 vaccines in African populations outside of South Africa. However, it does not thoroughly discuss the potential implications of this knowledge gap or the importance of generating more data in diverse geographical settings. Although the manuscript mentions that ChAdOx1 nCoV-19 is one of the most commonly used COVID-19 vaccines in low- and middle-income countries, it does not provide comprehensive information about the vaccine, its mechanism of action, or previous studies evaluating its safety and efficacy.

Results:

1. Lack of discussion on safety results: The manuscript briefly mentions adverse events but does not thoroughly analyse or discuss the safety data. It is important to discuss the nature, severity, and frequency of adverse events observed in the study population and any potential implications for vaccine safety.
2. Insufficient interpretation of immunogenicity results: While the manuscript mentions high seroconversion rates and strong immune responses following vaccination, it does not provide a comprehensive interpretation of these findings. It would be valuable to discuss the implications of these results regarding vaccine effectiveness, potential protection against SARS-CoV-2 infection, and the relevance of the immunogenicity data in the context of the global vaccine landscape.

Discussion:

1. Limited discussion of study limitations: The manuscript does not thoroughly discuss the study's limitations. It is important to address any potential biases, confounding factors, or limitations in the methodology that could impact the interpretation of the results. Acknowledging and discussing these limitations would provide a more balanced and informative discussion section.
2. Limited generalizability: The manuscript does not discuss the generalizability of the findings beyond the study population in coastal Kenya. Considering Africa's geographic and demographic diversity, it would be helpful to address the potential applicability of the results to other regions or people.
3. Inadequate conclusion: The manuscript does not provide a comprehensive discussion of the implications of the study findings for public health, vaccination strategies, or future research. It would be valuable to discuss how the results contribute to understanding COVID-19 vaccines in African populations and outline potential directions for future studies or interventions. The manuscript does not provide a concise conclusion summarising the key findings and their implications. A well-crafted conclusion is essential to reinforce the study's main message and offer a concise takeaway for readers.

Is the work clearly and accurately presented and does it cite the current literature?

Yes

If applicable, is the statistical analysis and its interpretation appropriate?

Yes

Are all the source data underlying the results available to ensure full reproducibility?

Yes

Is the study design appropriate and is the work technically sound?

Yes

Are the conclusions drawn adequately supported by the results?

Yes

Are sufficient details of methods and analysis provided to allow replication by others?

Yes

Reviewer Expertise:

Immunology and Microbiology

I confirm that I have read this submission and believe that I have an appropriate level of expertise to confirm that it is of an acceptable scientific standard, however I have significant reservations, as outlined above.

# Response

**Published:** 2023-11-06

**Authors:** George Warimwe

**Affiliations:** KEMRI-Wellcome Trust Research Programme, Kenya

This study contributes important data on the safety and immunogenicity of the ChAdOx1 nCoV-19 vaccine in an African population. It highlights the vaccine's ability to induce a strong immune response, as evidenced by high seroconversion rates and elevated IgG titres. The findings support using ChAdOx1 nCoV-19 in African countries and provide valuable evidence for vaccine deployment and decision-making.

 Overall, this study contributes significant knowledge on the ChAdOx1 nCoV-19 vaccine's safety and immunogenicity in an African population, supporting its use and providing insights for future research and vaccine implementation.

 The authors could improve the manuscript by addressing the following points:

 Introduction:

1. Lack of recent data: The manuscript references COVID-19 case and death statistics from December 2022, which is outdated considering the current date. Recent data should be used to represent the COVID-19 situation accurately.

*>We have updated the manuscript with the October 2023 data.*  

1. Inconsistency in COVID-19 severity: The introduction mentions that African populations have a significant clinical burden of SARS-CoV-2 exposure but many infected individuals show no clinical symptoms. However, it also states that hospitals have not shown substantial increases in admission rates. This contrast requires further clarification and supporting evidence.

 
*> We have removed the phrase “with a significant clinical burden”.  The statements about hospital admissions and asymptomatic infections were supported by references, and we agree these statements are not helped by the additional phrase.*

1. Limited data on vaccine safety and immunogenicity in Africa: The manuscript highlights the lack of clinical trial reports on the safety and immunogenicity of COVID-19 vaccines in African populations outside of South Africa. However, it does not thoroughly discuss the potential implications of this knowledge gap or the importance of generating more data in diverse geographical settings. Although the manuscript mentions that ChAdOx1 nCoV-19 is one of the most commonly used COVID-19 vaccines in low- and middle-income countries, it does not provide comprehensive information about the vaccine, its mechanism of action, or previous studies evaluating its safety and efficacy.

 
*> We add the following sentence on the requirement for these data:* *“In the absence of these data, decisions on use of the vaccine are based on generalizations of data from outside Africa and from South Africa alone.  Immunogenicity for other vaccines have varied with factors such as exposure to malaria [Ref -* *https://www.ncbi.nlm.nih.gov/pmc/articles/PMC4227280/* *], and adverse events have varied by population for other virally vectored vaccines [Ref -* *https://pubmed.ncbi.nlm.nih.gov/25830326/* *].  Data from Kenya are therefore needed to increase the database to provide confidence in public health decisions taken.”*

 Results:

1. Lack of discussion on safety results: The manuscript briefly mentions adverse events but does not thoroughly analyse or discuss the safety data. It is important to discuss the nature, severity, and frequency of adverse events observed in the study population and any potential implications for vaccine safety.

*> We have added the following in the discussion section;* *“* *The pattern of mild to moderate adverse events were not different to those seen for the licensed vaccine used as comparator (i.e. the rabies vaccine), and given the lack of severe or serious adverse events do not appear to be limiting.  Severe adverse events have been reported in larger studies, including thrombotic events (DOI: 10.1056/NEJMoa2104840) and myositis (* *https://doi.org/10.1016/S2665-9913(21)00312-X)* *but the study in Kenya was underpowered to detect rare events.  Ongoing pharmacovigilance is required, however the data seen in Kenya do not suggest a difference adverse event profile from that seen elsewhere.”*

1. Insufficient interpretation of immunogenicity results: While the manuscript mentions high seroconversion rates and strong immune responses following vaccination, it does not provide a comprehensive interpretation of these findings. It would be valuable to discuss the implications of these results regarding vaccine effectiveness, potential protection against SARS-CoV-2 infection, and the relevance of the immunogenicity data in the context of the global vaccine landscape.

*We have added further discussion on the T cell immune response in the fourth paragraph of the Discussion section as follows: “* *Furthermore, T cell responses were acquired during follow up among Rabies control vaccinees. These T cell responses are likely to be acquired as a result of SARS-CoV-2 transmission in the population, and initial T cell responses prior to vaccination may also be a result of cross-reactivity following circulation of endemic coronaviruses in Kenya (* *https://wellcomeopenresearch.org/articles/5-150* *). These responses in the control group would further dilute the impression of immunogenicity among ChAdOx1 vaccinees.* *”* *In the same paragraph, we highlight the similarity in the range of antibody levels observed in the Kenyan population as compared to populations in the UK, and that “Though our study was underpowered for efficacy assessment, the efficacy observed against asymptomatic infection was within the range reported in other studies” (preceding paragraph).  We believe this is contextualizes the observed immunogenicity and efficacy data to the global situation.*

 Discussion:

1. Limited discussion of study limitations: The manuscript does not thoroughly discuss the study's limitations. It is important to address any potential biases, confounding factors, or limitations in the methodology that could impact the interpretation of the results. Acknowledging and discussing these limitations would provide a more balanced and informative discussion section.

 
*We included the following discussion on limitations “Our study had several limitations. First, we predominantly recruited male participants. This is likely primarily due to the study requirement for long-term contraception which goes against cultural norms. Second, due to the prevailing patterns of health seeking behaviour we could not ascertain whether the upper respiratory tract infections reported during follow-up were COVID-19 cases, and there were no symptomatic episodes in any case. Third, due to the long duration of recruitment, trial participants were inevitably exposed to SARS-CoV-2, compromising analysis of vaccine-specific immune responses. This was evident in the gradual increase of anti-spike IgG titres and cellular responses in the rabies vaccine group over time.”* *We do not believe that bias or confounding factors are relevant given that the study was randomized.*

1. Limited generalizability: The manuscript does not discuss the generalizability of the findings beyond the study population in coastal Kenya. Considering Africa's geographic and demographic diversity, it would be helpful to address the potential applicability of the results to other regions or people.

 
*We add the following sentence:* *“While a single community in coastal Kenya cannot be considered representative of the diversity of the entire continent, taking these data in combination with the South African and global data strengthens our confidence in the safety and immunogenicity of the vaccine.”*

1. Inadequate conclusion: The manuscript does not provide a comprehensive discussion of the implications of the study findings for public health, vaccination strategies, or future research. It would be valuable to discuss how the results contribute to understanding COVID-19 vaccines in African populations and outline potential directions for future studies or interventions. The manuscript does not provide a concise conclusion summarising the key findings and their implications. A well-crafted conclusion is essential to reinforce the study's main message and offer a concise takeaway for readers.

 
*The full conclusion section now reads “* *While a single community in coastal Kenya cannot be considered representative of the diversity of the entire continent, taking these data in combination with the South African and global data strengthens our confidence in the safety and immunogenicity of the vaccine.* *We conclude that the vaccine is safe and immunogenic in Kenya, and suitable for ongoing use, although policy recommendations will need to also take account of ongoing data regarding the public health burden associated with the pandemic and cost effectiveness data (ref* *https://pubmed.ncbi.nlm.nih.gov/35914832/* *)”*
